# Phytochemical Profile, Antioxidant Capacity, α-Amylase and α-Glucosidase Inhibitory Potential of Wild Moroccan *Inula viscosa* (L.) *Aiton* Leaves

**DOI:** 10.3390/molecules26113134

**Published:** 2021-05-24

**Authors:** Fadoua Asraoui, Ayoub Kounnoun, Francesco Cacciola, Fouad El Mansouri, Imad Kabach, Yassine Oulad El Majdoub, Filippo Alibrando, Katia Arena, Emanuela Trovato, Luigi Mondello, Adnane Louajri

**Affiliations:** 1 Laboratory of Applied Biology and Pathology, Department of Biology, Faculty of Sciences of Tetouan, Abdelmalek Essaâdi University, Tetouan 93000, Morocco; a.kounnoun@gmail.com (A.K.); alouajri@hotmail.com (A.L.); 2 Department of Biomedical, Dental, Morphological and Functional Imaging Sciences, University of Messina, 98125 Messina, Italy; 3 Laboratory of Chemical Engineering and Valorization of Resources, Department of Chemistry, Faculty of Sciences and Technology, Abdelmalek Essaâdi University, Tangier 416, Morocco; fouad.elmansouri@etu.uae.ac.ma; 4 Laboratory of Biochemistry and Molecular Genetics, Faculty of Sciences and Technologies of Tangier, Tangier 416, Morocco; imad.kabach@gmail.com; 5 Department of Chemical Biological, Pharmaceutical and Environmental Sciences, University of Messina, 98168 Messina, Italy; youladelmajdoub@unime.it (Y.O.E.M.); arenak@unime.it (K.A.); lmondello@unime.it (L.M.); 6 Chromaleont s.r.l., c/o Department of Chemical, Biological, Pharmaceutical and Environmental Sciences, University of Messina, 98168 Messina, Italy; filippo.alibrando@chromaleont.it (F.A.); emanuela.trovato@chromaleont.it (E.T.); 7 Department of Sciences and Technologies for Human and Environment, University Campus Bio-Medico of Rome, 00128 Rome, Italy; 8 BeSep s.r.l., c/o Department of Chemical, Biological, Pharmaceutical and Environmental Sciences, University of Messina, 98168 Messina, Italy

**Keywords:** *Inula viscosa* (L.) *Aiton*, polyphenolic compounds, flavonoids, HPLC-DAD/ESI-MS, GC-MS, α-amylase, α-glucosidase, antioxidant activity

## Abstract

Medicinal plants offer imperative sources of innovative chemical substances with important potential therapeutic effects. Among them, the members of the genus *Inula* have been widely used in traditional medicine for the treatment of several diseases. The present study investigated the antioxidant (DPPH, ABTS and FRAP assays) and the in vitro anti-hyperglycemic potential of aerial parts of *Inula viscosa* (L.) *Aiton* (*I. viscosa*) extracts through the inhibition of digestive enzymes (α-amylase and α-glucosidase), responsible of the digestion of poly and oligosaccharides. The polyphenolic profile of the *Inula viscosa* (L.) *Aiton* EtOAc extract was also investigated using HPLC-DAD/ESI-MS analysis, whereas the volatile composition was elucidated by GC-MS. The chemical analysis resulted in the detection of twenty-one polyphenolic compounds, whereas the volatile profile highlighted the occurrence of forty-eight different compounds. *Inula viscosa* (L.) *Aiton* presented values as high as 87.2 ± 0.50 mg GAE/g and 78.6 ± 0.55mg CE/g, for gallic acid and catechin, respectively. The EtOAc extract exhibited the higher antioxidant activity compared to methanol and chloroform extracts in different tests with (IC_50_ = 0.6 ± 0.03 µg/mL; IC_50_ = 8.6 ± 0.08 µg/mL; 634.8 mg ± 1.45 AAE/g extract) in DPPH, ABTS and FRAP tests. Moreover, *Inula viscosa* (L.) *Aiton* leaves did show an important inhibitory effect against α-amylase and α-glucosidase. On the basis of the results achieved, such a species represents a promising traditional medicine, thanks to its remarkable content of functional bioactive compounds, thus opening new prospects for research and innovative phytopharmaceuticals developments.

## 1. Introduction

In the last years, the use of traditional medicine has meaningly expanded in the world, due to its effectiveness and minor side effects compared to synthetic drugs and, thus, selected medicinal herbs remedies have been employed, especially in less developed countries [1,2]. Morocco has a rich and ancient tradition in such a field and antique knowledge of medicinal plants has been used for therapeutical and nutritional purposes since long time [3]. Recently, there is a great interest by the Moroccan market in order to look for new and safe molecules capable to prevent and manage various diseases especially the ones related to free radical mechanism [4]. In addition to the use and development of synthetic drugs, different products have been obtained starting from plant species displaying many valuable effects on human health due to the great diversity of secondary metabolites, such as phenolic compounds, flavonoids, anthocyanins, carotenoids and vitamins [5].

In this context, the present study aimed to evaluate the potential of a traditionally used herbal medicine, *Inula viscosa* (L.) *Aiton* (*I. viscosa*) as an interesting source of antioxidant compounds. *I. viscosa* (L.) *Aiton* [*Dittrichia viscosa L. Greuter*] is an herbaceous perennial Mediterranean plant of the family *Asteraceae* [6]. It exhibits simple alternate leaves, characterized by glandular hairs, covered with glands secreting a sticky substance and bright yellow flowers that bloom between August and November [7]; it is widely distributed in Asia, Europe, Africa and predominant in the Mediterranean area, comprising of more than 100 species [8]. In Morocco, it is vernacularly known as “Bagramane” or “Magramane” and it has been employed topically, according to the traditional Pharmacopoeia to treat animal injuries. *I. viscosa* root and leaf decoctions have been used as useful and precious remedies for hypertension, diabetes mellitus and cardiac diseases [9,10]. Several recent experimental works have shown that extract of *I. viscosa* possess antifungal [11], antibacterial [12], hypoglycemic [13], antihypertensive [14], antiproliferative [15], anti-inflammatory [16] and strong antioxidant activity [17,18]. In addition, numerous secondary metabolites, isolated from *Inula* species, have shown their effectiveness against oxidative stress related diseases (cancer, diabetes and inflammation, etc.), as well as neurodegenerative disorders. For instance, alantolactone has been reported to be a polyvalent compound, displaying important bioactivities against these diseases [19]. Rutin has been reported to display good enzyme inhibitory properties, as well. In addition, other isolated compounds such as luteolin, nepitrin, nepetin, 3,5-*O*-dicaffeoylquinic acid, 1,5-*O*-dicaffeoylquinic acid, hispiduloside and jaceosidin have exhibited notable anti-inflammatory activity [20].

This work was designed to evaluate the total phenolics and flavonoids contents, the antioxidant properties (ABTS, DPPH and FRAP assays) and the potential inhibitory effects against key enzymes implicated in diabetes (α-amylase, α-glucosidase activities). In addition, the phytochemical profile, *viz.* polyphenols and volatile content of *I. viscosa* leaves extracts were determined by high-performance liquid chromatography coupled to photodiode array and electrospray ionization mass spectrometry (HPLC-DAD/ESI-MS) and gas chromatography coupled to mass spectrometry (GC-MS), respectively. 

## 2. Results and Discussion

### 2.1. Determination of Total Phenolics and Total Flavonoids Contents

The total phenolics and flavonoids contents (TPC and TFC) extracted from the leaves of *I. viscosa* are summarized in Table 1. In particular, the ethyl acetate (EtOAc) extract showed the highest TPC (87.2 ± 0.50 mg GAE/g of extract) and TFC (78.6 ± 0.55 mg CE/g of extract). On the other hand, the chloroform extract was found to be least rich in TPC (34.0 ± 0.48 mg GAE/g of extract) and TFC (18.3 ± 0.40 mg CE/g of extract). Such results are in agreement with previous studies where some variability among EtOAc, methanol and chloroform extracts was reported. The TPC values of Moroccan *I. viscosa* collected from the Taza region were higher than those reported by other authors [21] with values of 8.5 ± 1.04 mg GAE/g of extract and 2.6 ± 0.68 mg GAE/g of extract, for EtOAc and methanol leaves extracts, respectively. However, the results reported in reference [22], indicated a higher value of TPC (123.07 ± 1.69 mg GAE/g extract and lower value of TFC (30.9 ± 50 mg QE/g extract, for *I. viscosa* methanol extracts collected from Tunisia. In addition, a high value of TPC (274.4 ± 6.94 mg GAE/g DW) was attained for an EtOAc *I. viscosa* collected from the Sefrou region in Morocco [23]. The results of this study clearly indicate that the TPC and TFC of *I. viscosa* crude extracts vary according to the solvent extraction procedure and plant origin [24]. Additionally, numerous studies have shown the antioxidant capacity of plants to constructively correlate with TPC and TFC [25,26,27,28]. Phenolic acids and flavonoids are better extracted with hydrophilic solvents, whereas less hydrophilic ones such as chloroform may extract more lipophilic components such as triterpenoids that preferentially inhibits 5-lipoxygenase activity [29]. Indeed, multiple assays are usually recommended when determining the in vitro antioxidant capacity of plant samples [30]. 

### 2.2. Antioxidant Activity

The different extracts of *I. viscosa* were investigated for their antioxidant capacity using three complementary tests, DPPH, ABTS and FRAP (Table 2). The extracts showed an important antioxidant activity, especially the EtOAc extract with a value equal to (IC_50_ = 0.6 ± 0.03 µg/mL), which is close to the value obtained by the BHT used as positive control (IC_50_ = 0.38 ± 0.11 µg/mL); the methanol extract presented an IC_50_ value of 8.2 ± 1.16 µg/mL, whereas the chloroform extract exhibited an IC_50_ value equal to 40.8 ± 0.88 µg/mL. A similar trend was also observed for the ABTS test: the EtOAc extract yielded the highest antioxidant ability with a value of IC_50_ equal to 8.6 ± 0.15 µg/mL, compared to the methanol (IC_50_ = 25.5 ± 0.45 µg/mL) and chloroform (IC_50_ = 81.6 ± 0.05 µg/mL). Furthermore, in the FRAP method the highest reducing power was detected also in the EtOAc extract (634.8 ± 1.45 mg AAE/g extract), followed by methanol extract (552.1 ± 0.88 mg AAE/g extract) and chloroform extract with a value of 90.1 ± 0.66 mg AAE/g extract), In these assays, *I. viscosa* revealed interested antioxidant effects with slight variances among the tested extracts. *I. viscosa* EtOAc extract showed the highest antioxidant capacity compared with the reported results of Albano on the same plant from Portugal [29] with an IC_50_ = 3.6 µg/mL and Brahmi-Chendouh [31] with an IC_50_ = 14.1 ± 1.3 µg/mL for *I. viscosa* collected from Algeria. According to the results found by Mohti et al. [3] an IC_50_ of 148 µg ± 0.11 µg/mL (DPPH test) was attained for the methanolic extract of *Inula viscosa* (L.) *Aiton* leaves collected from Morocco. DPPH test. ABTS values for the extracts of the *Inula viscosa* (L.) *Aiton* investigated in this work are also greater compared to the values reported found in ref. [31] (IC_50_= 24.2 ± 1.0 µg/mL) and reference [32] (IC_50_= 16.7 ± 0.26 µg/mL) for methanolic leaves extracts. Hence, the relatively good radical scavenging ability demonstrated by the Inula species in the current work can be attributed to the polyphenolic compounds occurring in this plant contributing more effectively for the scavenging of free DPPH radicals. In fact, several secondary metabolites (rutin, quercitrin, quercetin, luteolin, kaempferol, isoquercitrin, chlorogenic acid, caffeic acid, β-caryophyllene and 1,3-dicaffeoylquinic acid) present in Inula species have been found to possess radical scavenging properties by DPPH and/or ABTS methods [19,33]. In addition, numerous other Inula species have been evidenced to exert antioxidant activity by radical scavenging property [32,34]. 

### 2.3. Enzyme Inhibitory Activities

Type 2 diabetes is a form of diabetes that is characterized by high blood sugar, insulin resistance and relative lack of insulin and it represents over 90% of diabetes cases worldwide. The decrease or inhibition of carbohydrate absorption by reducing digestive enzymes such as α-amylase and α-glucosidase is one of the highest widely used strategies to reduce postprandial hyperglycemia. In our study, *Inula viscosa* (L.) *Aiton* organic extracts were also tested for their inhibitory activities against the enzymes α-glucosidase and α-amylase. The α-glucosidase is a key intestinal enzyme in carbohydrate digestion. Inhibitors of α-glucosidase can postpone the uptake of dietary carbohydrates and suppress postprandial hyperglycemia. This can also lead to the reduction of oxidative damage, which is a key mechanism in insulin resistance [35]. The obtained results are listed in (Table 3). All extracts presented a higher effect of α-glucosidase inhibition compared than the standard drug acarbose (IC_50_ = 33.0 µg/mL). The latter is a potent inhibitor of α-glucosidase and α-amylase. Moreover, the methanolic extract of Inula. *I. viscosa* revealed the ultimate inhibition potential activity against α-glucosidase with an IC_50_ = 22.3 µg/mL; as highlighted in (Table 3), the percentage of the enzyme inhibition versus concentration of *I. viscosa* extract ranged from 333 μg/mL to 10 μg/mL. The difference in inhibitory effects among the three solvent of leaves extracts of *I. viscosa* is certainly due to the difference in chemical functional compounds extracted by each solvent. However, several side effects are associated to the consumption of acarbose. For instance, it incites diarrhea by disproportionate inhibition of the amylase enzyme in the gastrointestinal tract [36]. The excessive inhibition of pancreatic amylase can lead to abnormal bacterial fermentation of carbohydrate foods in the colon, which may lead to adverse digestive disorders [36,37]. Additional studies have described hepatotoxicity and hepatic injury [38] and elevation of liver enzyme levels [38] resulting from acarbose intake. In this context, medicinal plants may have high effectiveness and less harmful effects than existing drugs [39,40]. For this aim, studies are constantly performed to find alternatives source from medicinal plants as treatment for type 2 diabetes. The results achieved reveal the potential properties of *I. viscosa* to reduce the postprandial increase of blood glucose amounts in diabetic persons and their capacities to prevent type 2 of diabetes and attributes the antioxidant and the inhibitory activities of aerial part extracts to the phenolic and flavonoid contents of the plant. 

### 2.4. Phytochemical Profile of I. viscosa by GC-MS and HPLC-DAD/ESI-MS

The attained results of the GC-MS analysis of the *n*-hexane fraction of *I. viscosa* showed the presence of forty-eight compounds belonging to different chemical classes (Table 4). The % of similarity for the identified compounds ranged from 88 to 98%. Studies in rats have shown, *in vitro*, that cuminaldehyde has an inhibitory effect on the aldosereductase and α-glucosidase enzymes, leading the way to potential use as an antidiabetic agent [41]; on the other hand, α-Zingiberene, α-Cubebene, β-Cubebene, α-Curcumene, belonging to the sesquiterpenes class, have already demonstrated their antioxidant properties which might explain at some extent the results obtained in the present study [42].

**Figure 1:**
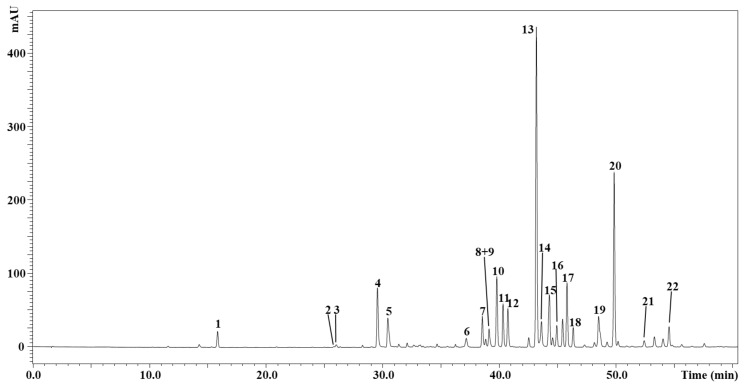
Polyphenolic profile of *I. viscosa* EtOAc extract by investigated by HPLC-DAD-ESI/MS (330 nm).

The polyphenolic profile of *I. viscosa* EtOAc extract, attained by HPLC-DAD-ESI/MS analysis, is displayed in Figure 1. Peak identification is reported in Table 5, where a total of 21 polyphenols were detected and 19 out of them were tentatively identified on the basis of retention times, MS and literature data [3,31,32,43,44]. Five of them are phenolic acids, namely caffeic acid, galloylquinic acid, two isomers of di-*O*-Caffeoylquinic acids and rosmarinic acid, whereas the rest is represented by flavonoids *viz.* derivatives of quercetin, luteolin, naringin and apigenin. Almost the totality of them have been previously reported as constituents of *I. viscosa* leaves [3,31,32,43,44] with the exception of peak no. 3 (Figure 1, Table 5), dihydroquercetin, which has never been reported before. As far as quantification is concerned, the most abundant compound was represented by diosmetin (3365.2 mg/Kg, peak no. 13), followed by rosmarinic acid (1529.5 mg/Kg, peak no. 20).

In general, the present polyphenolic profile, particularly rich in mono- and dicaffeoylquinic acids as well as flavonols and flavanones could, at least in part, explain the strong antioxidant and hypoglycemic activity especially of the EtOAc and methanolic extract of *I. viscosa* So far, previous studies have pinpointed the high ability of dicaffeoylquinic acid isomers to scavenge free radicals [18,45]. The presence of functional groups (hydroxyl and caffeoyl groups) in the structure of the identified phenolics is responsible for their strong antioxidant activity. Being polyphenolic compounds found in numerous medicinal plants and herbal drugs, these bioactive compounds have often been used in pharmacological applications [46]. In fact, they are known to possess a wide range of biological activities such as antioxidant, chemopreventive, anticancer, antimalarial and antidiabetic properties, [47,48,49,50,51,52].

### 2.5. Statistical Analysis

A paired difference test is a statistical technique that is used to compare the difference between the means obtained by each type of solvent. This analysis (Table 6) showed that there was a significant (*p* < 0.05) difference between the means of all the assays studied.

The statistical analysis (Table 7) shows a significant difference (*p* < 0.05) between the means obtained by each type of solvent in all the assays studied. EtOAc provided a better quality of the extract than the other solvents with a value of 8.63 ± 0.09 μg/mL, 0.62 ± 0.04 μg/mL and 634.81 ± 1.45 mg/g, respectively, at the level of the analysis of ABTS, DPPH and FRAP; on the other hand, the methanolic extract showed a better quality of extract compared to the other solvents with a value of 22.26 ± 2.82 μg/mL and 27.16 ± 1.6%, respectively, at the level of the analysis of α-glucosidase and α-amylase inhibition.

## 3. Materials and Methods

### 3.1. Plant Material

*Inula viscosa* (L.) *Aiton*, was collected from Taza region in Morocco, in spring season 2018 and dried approximately for 2 weeks at ambient temperature. Identification was confirmed by Professor Mohamed El kadiri, botanist at the faculty of sciences Tetouan, Morocco, and disposed at the herbarium of the laboratory with a voucher specimen code IV-LABP02. Plant material was dried in the shade at room temperature, powdered to achieve a mean particle size and kept in the dark until future analysis.

### 3.2. Chemical Reagents and Solvents

2,2-Diphenyl-1-picrylhydrazyl (DPPH), 2,20-azino-bis-(3-ethylbenzothiazoline-6-sulfonic) acid (ABTS), L-ascorbic acid and butylated hydroxytoluene (BHT), ρ-Nitrophenyl-α-D-glucopyranoside (pNPG), α-glucosidase, α-amylase, were purchased from Sigma (St. Louis, MO, USA). Folin-Ciocalteu phenol reagent and standards (gallic acid, kaempferol and quercetin) were obtained from Merck Life Science (Merck KGaA, Darmstadt, Germany). LC-MS grade methanol, acetonitrile, acetic acid, acetone and water were also purchased from Merck Life Science. All other chemicals were of analytical grade and obtained from Sigma.

### 3.3. Preparation of Crude Extracts

The extraction of samples was conducted by Soxhlet apparatus with methanol, ethyl acetate and chloroform, to get three extracts with different polarities. A total of 50 g of dried leaves of *I. viscosa* were rigorously extracted with 250 mL of each solvent, then the obtained extracts were concentrated and free of solvent under reduce pressure, using rotary evaporator then evaporated to dryness. At the end of the extraction operation three crude extracts were obtained and were subsequently weighed to calculate the yield of the extraction for each solvent and stored in a refrigerator (4 °C) in airtight bottles until used for further analysis. For GC-MS analysis, five grams of plant powder was defatted three times in 50 mL of *n*-hexane and the extraction was performed by sonication in an ultrasound bath (130 kHz) for 45 min. After centrifugation for 5 min, the supernatant was filtered through a paper filter, dried with rotary evaporator and reconstituted with *n*-hexane, prior to GC-MS analysis.

### 3.4. Analysis and Quantification of Phenolic Contents

Total phenol content of *I. viscosa* leaves extracts was determined by a spectrophotometric method using the Folin–Ciocalteu reagent according to reference [53] with some modification, for determination 100 µL of Folin-Ciocalteu reagent solution and the reaction mixture was basified by adding 400 µL of 7.5% sodium carbonate (Na_2_CO_3_); then, the mixture was homogenized and kept in the dark for 60 min at room temperature, the absorbance was measured at 765 nm. The TPC in samples was quantified from a calibration curve prepared with gallic acid standard with different concentrations and expressed as mg of gallic acid equivalents (GAE) per g of extract (mg GAE/g extract) of the sample.

Total flavonoid content of *I. viscosa* leaves extract was determined by a spectrophotometric method using the aluminum chloride (AlCl_3_) based on the protocol described by ref. [54] with slight modifications. Briefly, (0.5 mL) of the extract solution mixed with 1.5 mL of 80% methanol, 0.1 mL of 10% aluminum chloride, 0.1 mL of 1 M potassium acetate (CH_3_COOK) and 2.8 mL of deionized water. After incubation at room temperature for 30 min, the absorbance of the reaction mixture was measured at 415 nm against deionized water blank. The TFC in samples was quantified from a calibration curve prepared with catechin standard with different concentrations and expressed as mg of catechin equivalents (CE) per g of extract (mg CE/g extract) of the sample.

### 3.5. Determination of Antioxidant Capacity

#### 3.5.1. Scavenging Capacity of DPPH Radical

Free radical scavenging activity of antioxidants was estimated by the method described by ref. [55], slightly modified, using DPPH (2,2-diphenyl-1-picrylhydrazyl), a stable free radical, different concentration of samples prepared in methanol solution and 500 µL of 0.2 mM of DPPH methanolic solution was added to the mixture and it was vortexed thoroughly, after 30 min incubation time in darkness at room temperature the absorbance was measured at 517 nm, with a blank containing DPPH and methanol. The butylated hydroxytoluene (BHT) was used as a standard. DPPH scavenging activity was expressed as the concentration of sample necessary to give a 50% reduction in the sample absorbance (IC_50_).

#### 3.5.2. Scavenging Capacity of ABTS Radical Cation

The total antioxidant capacity of the components was measured by the (2,20-azino-bis-(3-ethylbenzothiazoline-6-sulfonic acid) ABTS decolorization assay involving preformed ABTS+ radical cations, as described previously by ref. [56]. The ABTS stock solution was produced by reacting ABTS aqueous solution (7 mM) with potassium persulfate aqueous solution (2.45 mM), the mixture was kept in the dark at room temperature for 12–16 h, then the ABTS^+^ stock solution was diluted with methanol to an absorbance of 0.7 ± 0.02 at 734 nm, 3.9 mL of the ABTS+ solution was mixed with 0.1 mL of test sample diluted at different concentrations, then the mixture was incubated for 10 min in the dark, ascorbic acid was used as a standard, the absorbance of the resulting solution was measured at 734 nm. ABTS radical scavenging activity was expressed as the concentration of sample necessary to give a 50% reduction in the sample absorbance (IC_50_).

#### 3.5.3. Total Reducing Power Assay Fe (III) to Fe (II)

The capacity of *I. viscosa* extracts to reduce iron (III) to iron (II) was evaluated according to the method reported in reference [57] and slightly modified by reference [58]. Briefly, 1 mL of the sample test mixed with 2.5 mL of phosphate buffer (0.2 M, pH 6.6) and 2.5 mL of potassium hexacyanoferrate III (1%), after 30 min of incubation at 50 °C, 2.5 mL of trichloroacetic acid (10%) were added, then the mixture was centrifuged for 10 min at 3000 rpm. Finally, the supernatant fractions (2.5 mL) were mixed with distilled water (2.5 mL) and FeCl_3_ (0.1 mL, 0.1%). The absorbance of the resulting solution was measured at 700 nm. Reducing power was expressed in relation to the reducing power of ascorbic acid, as a positive control ascorbate equivalent antioxidant capacity (AEAC) (mg AAE/g DE).

### 3.6. Enzyme Inhibitory Activities

#### 3.6.1. α-Glucosidase Inhibition Assay

The α-glucosidase inhibitory activity was achieved in PBS (0.1 M KH_2_PO_4_–K_2_HPO_4_, pH 6.7), using ρ-nitrophenyl-α-D-glucopyranoside (ρNPG) as a substrate according to the method described by reference [59] with some modifications. Generally, all tested extracts were dissolved in PBS to a series of different concentrations. Briefly, a mixture of 165 μL of the samples and 110 μL of PBS containing the enzyme α-glucosidase solution (0.1 U/mL) were incubated at 37 °C for 10 min. Then, 220 μL ρ-nitrophenyl-α-D-glucopyranoside (1 mM) were added to the mixture to initiate the reaction. After further incubation at 37 °C for 30 min, Then, the reaction was terminated by the addition 605 µL of sodium carbonate solution. Na_2_CO_3_ (0.1 M) and the absorbance was measured at 405 nm. Acarbose was used as a standard inhibitor. The inhibition effect was calculated as follows: % α-glucosidase Inhibition = (absorbance of negative control-absorbance of sample)/absorbance of negative control) ×100. The IC_50_ value indicates the effective concentration that could inhibit 50% of glucosidase activity.

#### 3.6.2. α-Amylase Inhibition Assay

The α-amylase inhibitory potential was performed by reacting different concentrations of extracts with α-amylase and starch solution, as described by reference [60] with some modifications. Sample’s solution (250 μL) was mixed with 250 μL of 0.02 M PBS (pH 6.9) containing the α-amylase enzyme (240 U/mL) and incubated for 20 min at 37 °C. Soluble starch (1%, PBS 0.02, pH 6.9) was added to the mixture and further incubated at 37 °C for 20 min. The reaction was stopped by adding 250 μL of dinitrosalicylic acid and the incubation of the solution at 90 °C in a water bath for 10 min. The cooled reaction mixture was diluted with 1 mL deionized water and the absorbance was measured at 540 nm. The α-amylase inhibitory activity was expressed as a percentage of inhibition.

### 3.7. GC-MS

The analysis of the volatile fraction was carried out on a GC-MS-QP2020 system (Shimadzu, Kyoto, Japan) with an “AOC-20i” system auto-injector. The chromatographic column was an SLB-5ms column (30 m in length × 0.25 mm in diameter × 0.25 μm in thickness of film, Merck Life Science, Merck KGaA, Darmstadt, Germany). The initial temperature was set at 50 °C, afterwards increased up to 350 °C (increase rate: 3 °C/min; holding time: 5 min). GC-MS parameters were as follows: injection temperature: 280 °C; injection volume: 0.3 μL (split ratio: 10:1); pure helium gas (99.9%); linear velocity: 30.0 cm/s; Inlet pressure: 26.7 KPa. EI source temperature: 220 °C; Interface temperature: 250 °C. The acquisition of MS spectra was carried out in full scan mode, in the mass range of 40–660 m/z, with an event time of 0.2 s. Relative quantity of the chemical compounds present in each sample was expressed as percentage based on peak area produced in the GC chromatogram.

Compounds were identified by using the “FFNSC 4.0” (Shimadzu Europa GmbH, Duisburg, Germany) and “W11N17” (Wiley11-Nist17, Wiley, Hoboken, NJ, USA; Mass Finder 3). Each compound was identified applying a MS similarity match and an LRI filter. Linear retention indices (LRI) were calculated by using a C7-C40 saturated alkanes reference mixture (49452-U, MerckLifeScience, MerckKGaA, Darmstadt, Germany). Data files were collected and processed by using “GCMS Solution” software, ver. 4.50 (Shimadzu, Kyoto, Japan).

### 3.8. HPLC-DAD/ESI-MS

The LC analysis of the polyphenolic content was carried out on a Shimadzu liquid chromatography system (Kyoto, Japan) consisting of a CBM-20A controller, two LC-20AD dual-plunger parallel-flow pumps, a DGU-20A5R degasser, a SIL-20AC autosampler, an SPD-M30A photo diode array detector and an LCMS-8050 triple quadrupole mass spectrometer, through an ESI source (Shimadzu, Kyoto, Japan).

Chromatographic separations were performed on 150 mm × 4.6 mm; 2.7 µm Ascentis Express RP C18 column (Merck Life Science, Merck KGaA, Darmstadt, Germany). The mobile phase was composed of two solvents: water/acetic acid (99.85/0.15 *v/v*, solvent A) and acetonitrile/acetic acid (99.85/0.15 *v/v*, solvent B), The flow rate was fixed at 1 mL/min under gradient elution: 0-5 min, 5% B, 5–15 min, 10% B, 15–30 min, 20% B, 30–60 min, 50% B, 60 min, 100% B. DAD detection was applied in the range of λ = 200–400 nm and a wavelength of 280 nm was monitored (sampling frequency: 40.0 Hz, time constant: 0.08 s). MS conditions were as follows: scan range and scan speed were set at m/z 100–800 and 2500 u sec^−1^, respectively, event time: 0.3 sec, nebulizing gas (N_2_) flow rate: 1.5 L min^−1^, drying gas (N_2_) flow rate: 15 L min^−1^, interface temperature: 350 °C, heat block temperature: 300 °C, DL (desolvation line) temperature: 300 °C, DL voltage: 1 V, interface voltage: −4.5 kV. Calibration curves (R^2^ ≥ 0.997) of seven polyphenolic standards were used for the quantification of the EtOAc extract.

### 3.9. Statistical Analysis

All data were analysed using IBM SPSS Statistics for Windows, version 21 (IBM Corp., Armonk, NY, USA). The experiments were carried out in triplicates and the results are expressed as the average of the three measurements ±SD. The comparison of means between analysis study was performed with one-way analysis of variance (ANOVA). Differences were considered significant when *p* < 0.05.

## 4. Conclusions

The present study investigated the polyphenolic content, antioxidant activity and en-zymes inhibitory activities of EtOAc, methanol and chloroform extracts of *I. viscosa*, obtained using Soxhlet extraction method. The extracts (especially the EtOAc one) showed a considerable antioxidant effect against the DPPH, ABTS and the ferric reducing power FRAP assays. Moreover, they showed an important inhibitory capacity against the enzymes α-amylase and α-glucosidase compared to the standard synthetic compounds. These results suggest that the polar extracts from this Mediterranean and underused species from Morocco can be useful in therapeutic side due to its remarkable antioxidant and antidiabetic abilities. The antidiabetic effects are related to the inhibition of enzymes im-plicated in sugar metabolism. Moreover, antioxidant effects of *I. viscosa* can also be beneficial to improve the management of people with diabetes. The obtained results of this study reveal the potential application use of *I. viscosa* crude ex-tracts in the field of pharmaceutical industries, in particularly as antioxidant and antihyperglycemic treatment. However, further investigations regarding the isolation of these main compounds and evaluation of their antioxidant and antidiabetic activities.

## Figures and Tables

**Table 1:** TPC and TFC of *I. viscosa* leaves extracts. Data are expressed as mean ± SD (*n* = 3).

Extracts	Polyphenols (mg GAE/g of Extract)	Flavonoids (mg CE/g of Extract)
EtOAc	87.2 ± 0.50	78.6 ± 0.55
Methanol	65.3 ± 0.78	52.1 ± 0.80
Chloroform	34.0 ± 0.48	18.3 ± 0.40

mg GAE/g extract: mg gallic acid equivalents per g of extract. mg CE/g extract: mg of catechin equivalent per g of extract.

**Table 2:** Antioxidant activity of *Inula viscosa* (L.) *Aiton* extracts.

Antioxidant Properties (IC_50_ Value µg/mL ± Standard Deviation)			
Plant Extracts	DPPH	ABTS	FRAP (mg EAA/g DW)
EtOAC	0.6 ± 0.03	8.6 ± 0.08	634.8 ± 1.45
Methanol	8.2 ± 1.16	25.5 ± 0.45	552.1 ± 0.88
Chloroform	40.8 ± 0.88	81.6 ± 0.05	90.1 ± 0.66
BHT	0.3 ± 0.11	-	-
Ascorbic acid	-	16.9 ± 4.77	-

**Table 3:** Digestive enzymes inhibition activity (α-glucosidase and α-amylase) of *I. viscosa* extracts expressed in IC_50_ and percentage of inhibition (%). Data are expressed as mean ± SD (*n* = 3).

Plant Extracts	IC_50_ (µg/mL)	Percentage of Inhibition (%)
	α-Glucosidase Inhibition	α-Amylase Inhibition
EtOAc	29.9 ± 1.04	22%
Methanol	22.3 ± 2.82	27%
Chloroform	39.8 ± 0.76	17%

**Table 4:** List of compounds identified in the n-hexane fraction of *I. viscosa* by GC-MS.

#	Compounds	Match	LRI Ref	LRI Exp	Library
1	Cuminaldehyde	98	1243	1246	FFNSC 4.0
2	Phenylacetic acid	95	1261	1251	FFNSC 4.0
3	α-Terpinen-7-al	97	1287	1290	FFNSC 4.0
4	α-Cubebene	96	1347	1348	FFNSC 4.0
5	Eugenol	94	1357	1357	FFNSC 4.0
6	α-Copaene	96	1375	1377	FFNSC 4.0
7	β-Cubebene	93	1392	1389	FFNSC 4.0
8	(*E*)-Caryophyllene	97	1424	1421	FFNSC 4.0
9	Germacrene D	92	1478	1477	FFNSC 4.0
10	α-Curcumene	93	1480	1482	FFNSC 4.0
11	β-Selinene	91	1492	1491	FFNSC 4.0
12	α-Zingiberene	92	1496	1496	FFNSC 4.0
13	α-Muurolene	96	1497	1501	FFNSC 4.0
14	(*E*,*E*)-, α-Farnesene	89	1504	1505	FFNSC 4.0
15	epi-Cubebol	89	1498	1506	FFNSC 4.0
16	β-Bisabolene	97	1508	1509	FFNSC 4.0
17	γ-Cadinene	96	1512	1516	FFNSC 4.0
18	δ-Cadinene	92	1518	1521	FFNSC 4.0
19	β-Sesquiphellandrene	96	1523	1526	FFNSC 4.0
20	α-Cadinene	94	1538	1540	FFNSC 4.0
21	Caryophyllene oxide	94	1587	1586	FFNSC 4.0
22	Fokienol	97	1596	1601	FFNSC 4.0
23	β-Oplopenone	88	1606	1608	FFNSC 4.0
24	δ-Cadinol	92	1641	1650	FFNSC 4.0
25	α-, epi-Muurolol	93	1645	1654	FFNSC 4.0
26	Cadin-4-en-10-ol	92	1659	1665	FFNSC 4.0
27	Oplopanone	92	1738	1744	FFNSC 4.0
28	Neophytadiene	95	1836	1837	FFNSC 4.0
29	Phytone	92	1841	1843	FFNSC 4.0
30	*n*-Hexadecanoic acid	94	1977	1977	FFNSC 4.0
31	(*Z*,*Z*)-9,12-Octadecadienoic acid	92	2140	2142	W11N17
32	(*Z*,*Z*,*Z*)-9,12,15-Octadecatrienoic acid	97	2154	2152	W11N17
33	*n*-Tricosane	97	2300	2300	FFNSC 4.0
34	*n*-Tetracosane	97	2400	2400	FFNSC 4.0
35	*n*-Pentacosane	97	2500	2501	FFNSC 4.0
36	*n*-Hexacosane	95	2600	2600	FFNSC 4.0
37	*n*-Heptacosane	96	2700	2701	FFNSC 4.0
38	methyl-Tetracosanoate	96	2732	2733	FFNSC 4.0
39	*n*-Octacosane	94	2800	2800	FFNSC 4.0
40	2-methyl-Octacosane	95	2864	2863	W11N17
41	*n*-Nonacosane	92	2900	2901	FFNSC 4.0
42	Methyl hexacosanoate	93	2940	2935	W11N17
43	*n*-Triacontane	93	3000	3000	FFNSC 4.0
44	*n*-Hentriacontane	95	3100	3101	FFNSC 4.0
45	*n*-Dotriacontane	94	3200	3200	FFNSC 4.0
46	*n*-Tritriacontane	94	3300	3300	FFNSC 4.0
47	*n*-Tetratriacontane	92	3400	3400	FFNSC 4.0
48	*n*-Pentatriacontane	91	3500	3500	FFNSC 4.0

**Table 5:** Polyphenolic compounds detected in *I. viscosa* EtOAc extract by HPLC-DAD/ESI-MS.

*N*	Compounds	t_R_ (min)	UV_max_ (nm)	[M-H]^-^	Content (mg/kg)	Employed Standard for Quantification	Ref.
1	Caffeic acid	15.83	322	179	157.7 ± 0.13	Caffeic acid	[41]
2	Galloylquinic acid	25.82	281	343	80.9 ± 0.18	Gallic acid	[42]
3	Dihydroquercetin	26.02	287	303	119.6 ± 0.12	Quercetin	-
4	Di-*O*-Caffeoylquinic acid	29.55	328	515	621.0 ± 1.53	Caffeic acid	[42]
5	Di-*O*-Caffeoylquinic acid isomer	30.45	326	515	374.8 ± 1.27	Caffeic acid	[42]
6	Unknown	37.18	288	181	-	-	-
7	Quercetin	38.55	359	301	384.7 ± 0.94	Quercetin	[42]
8	Nepetin	39.14	342	315	191.8 ± 0.14	Luteolin	[42]
9	Padmatin	39.22	290	317	573.5 ± 2.15	Naringin	[42]
10	Unknown	39.79	289	635, 317	-	-	-
11	3-*O*-methylquercetin	40.33	355	315	486.5 ± 1.18	Quercetin	[3]
12	Spinacetin	40.76	291, 339 sh	345	450.6 ± 1.44	Apigenin	[42]
13	Diosmetin	43.18	273 sh, 335	299	3365.2 ± 4.32	Apigenin	[42]
14	Rhamnetin	43.60	355	315	502.1 ± 1.77	Luteolin	[42]
15	Hesperetin	44.30	290	301	660.3 ± 0.36	Naringin	[3,42]
16	Hispidulin	44.92	348	299	281.4 ± 0.14	Apigenin	[3,42,44]
17	Cirsiliol	45.44	339	329	313.1 ± 0.29	Apigenin	[42]
18	3-*O*-Acetylpadmatin	46.34	350	359	683.3 ± 0.36	Naringin	[42]
19	Isorhamnetin	48.52	367	315	820.3 ± 1.77	Luteolin	[42]
20	Rosmarinic acid	49.86	323	359	1529.5 ± 0.99	Rosmarinic acid	[42]
21	Luteolin	52.43	288	285	82.3 ± 0.30	Luteolin	[3,43]
22	Unknown	54.50	292	343	-	-	-

Values are expressed as the mean ± S.D. (*n* = 3), sh: Shoulder.

**Table 6:** Statistical analysis performed by a paired difference test.

Paired Difference Values								
Type of Analysis	Type of Solvent	Mean	Ecart Type	Variance	Std. Error	95% Confidence Interval of Difference		Sig. (Bilateral)
						Lower Bound	Upper Bound	
Polyphenols (mg GAE/g of extract)	EtOAc-Methanol	21.900	0.100	0.010	0.057	21.651	22.148	0.000 S
	EtOAc-Chloroform	53.200	0.150	0.0225	0.086	52.827	53.572	0.001
	Methanol-Chloroform	31.300	0.050	0.0025	0.028	31.175	31.421	0.000 S
Flavonoids (mg CE/g of extract)	EtOAc-Methanol	26.500	0.100	0.01	0.057	26.251	26.748	0.001 S
	EtOAc-Chloroform	60.300	0.086	0.0073	0.050	60.084	60.515	0.000 S
	Methanol-Chloroform	33.800	0.050	0.0025	0.028	33.675	33.924	0.000 S

S: Significant (*p* < 0.05). Values are averages ± standard deviation of triplicate analysis.

**Table 7:** Statistical analysis of the means performed by one-way analysis of variance (ANOVA).

Type of Analysis	Solvant Type	Average	Ecart Type	95% Confidence Interval		Test ANOVA	
				Lower Bound	Upper Bound	Variance	Sig.
ABTS assay (IC_50_ μg/mL)	EtOAc	8.633 ± 0.088	0.152	8.254	9.013	0.023	0.001 S
	Chloroform	81.646 ± 0.057	0.100	81.3978	81.895	0.010	
	Methanol	25.223 ± 0.453	0.785	23.2711	27.175	0.618	
FRAP assay (mg/g)	EtOAc	634.810 ± 1.452	2.516	628.558	641.062	6.333	0.000 S
	Chloroform	90.143 ± 0.666	1.154	87.275	93.0123	1.333	
	Methanol	552.143 ± 0.881	1.527	548.349	555.938	2.333	
DPPH assay (IC_50_ μg/mL)	EtOAc	0.62 ± 0.037	0.064	0.46	0.78	0.004	0.000 S
	Chloroform	40.85 ± 0.887	1.536	37.03	44.66	2.360	
	Methanol	8.17 ± 1.165	2.018	3.16	13.19	4.073	
α-glucosidase inhibition assay (IC_50_ μg/mL)	EtOAc	29.920 ± 1.049	1.817	25.405	34.436	3.305	0.000 S
	Chloroform	39.801 ± 0.768	1.330	36.497	43.106	1.770	
	Methanol	22.263 ± 2.825	4.894	10.104	34.422	23.957	
α-amylase inhibition assay (%)	EtOAc	22.152 ± 0.387	0.670	20.486	23.819	0.450	0.000 S
	Chloroform	17.157 ± 0.634	1.099	14.426	19.887	1.208	
	Methanol	27.162 ± 1.623	2.811	20.178	34.146	7.904	

All values were significant (*p* < 0.05). Values are averages ± standard deviation of triplicate analysis. Data obtained were subjected to one-way Analysis of Variance (ANOVA).

## Data Availability

Not applicable.

## Sample Availability

Samples of the compounds are not available from the authors.
